# Spatial and non-verbal reasoning abilities in first-year female DVM students before and after 4 h of canine osteology training or 19 h of canine dissection: preliminary study

**DOI:** 10.3389/fvets.2025.1593360

**Published:** 2025-07-21

**Authors:** J. Claudio Gutierrez, Uchenna Nlebedum, Patrawin Wanakumjorn, Steven D. Holladay

**Affiliations:** ^1^School of Veterinary Medicine, University of California, Davis, Davis, CA, United States; ^2^College of Veterinary Medicine, University of Georgia, Athens, GA, United States

**Keywords:** spatial ability, anatomy, visual reasoning, non-verbal reasoning, logical reasoning

## Abstract

Spatial ability refers to human cognitive ability to form, retrieve, and mentally manipulate models of spatial nature. This critical component of human intellect is relevant on a wide spectrum of professional disciplines including engineering, architecture, mathematics, computer sciences, natural sciences and a variety of medical disciplines, including anatomy and diagnostic imaging. In the present study, validated testing tools were used to compare spatial and general non-verbal reasoning abilities in first-year female veterinary medical students. These tests were: Guay’s Visualization of Views Test (GVVT) and, Raven’s Advanced Progressive Matrices Test, short form (APMT). Osteology Group (OG): students took the tests before and after exposure to general canine osteology (4 h). Dissection Group (DG): students took the tests before and after exposure to dissections/pro-sections/palpation labs (19 h). Results for the OG showed a numeric but non-significant increase in GVVT (*p* = 0.092), with mean scores of 8.01 and 11.34 pre-training and post-training, respectively. Similar results were found for the APMT, with pre-training and post-training mean scores of 7.44 and 8.44 (*p* = 0.16), respectively. Results for the DG showed a numeric but non-significant increase in GVVT (*p* = 0.67), with mean scores of 11.77 and 13.28 pre-labs and post-labs, respectively. For the APMT, the increase in scores was significant (*p* = 0.028), with mean scores of 6.8 and 10.2, pre-labs and post-labs, respectively. Future studies are planned with greater numbers of students and groups with different hours of anatomy exposure. Future studies might also consider subgroups such as pre-veterinary students.

## Introduction

Spatial ability is a critical component of the human intellect that differs widely by individuals. This cognitive ability relates to the process by which internal three-dimensional representations of objects are mentally generated based on the assimilation and integration of a series of two-dimensional spatial displays. Such ability concerns three-dimensional (3D) structure and position of objects when they are being manipulated, or mental transformation of object representations and correct recognition of 3D objects viewed from different positions (1). It is relevant on a wide spectrum of professional disciplines, including engineering, architecture, mathematics, computer sciences, natural sciences, and different medical disciplines. Exposure of students to different curricula has been found to enhance spatial intelligence, suggesting learning may be enhanced if greater attention is given to the special aspects of concepts being presented (e.g., through greater implementation of visual aids such as pictures and graphs, diagrams and discipline-relevant models). In short, while audible teaching methods are of time-proven high value, they may also not capture to the full potential the fundamental ideas educators are striving to convey (2, 3).

There are different components of spatial ability and well-established tests for measuring such aspects. Guay’s visualization of views test, adapted version (GVVT) is one such widely-used standard test to measure spatial ability (4). Tests of spatial ability can complement standardized general non-verbal reasoning tests that employ a series of perceptual analytical/logical problems, each in the form of a matrix. Raven’s advanced progressive matrices test, short form (APMT), is one such test of general reasoning ability that can be used as a complement to the GVVT (2, 3, 5, 6). In addition, correlation between general non-verbal reasoning ability and spatial ability has been previously reported (3).

Students enrolled in accredited human medical programs were found to possess higher spatial ability than students enrolled in other science disciplines, and to also show spatial ability improvement with progression through the medical curriculum (7, 8). Related studies in veterinary medical students found increases in spatial ability scores at both 32 and 64 weeks into the Doctor of Veterinary Medicine (DVM) curriculum as compared to student entry level scores (2, 9). These authors suggested the experiencing of substantial anatomic dissection, an intense, highly visual and 3-dimensional portion of the curriculum, may play a role in the increase of spatial skills. At the undergraduate level, Guillot et al. similarly found significant positive correlation between spatial ability and academic outcome in anatomy courses (7). These same authors also reported increased spatial ability as undergraduate students progressed through curricula that included anatomy courses. Although several such authors have suggested the anatomy portion of the curriculum was either partially or most responsible for the improved spatial ability scores, attempts have not yet been made to separate anatomy training from the rest of the curricular experiences.

In a different field, short periods of training using video games have been shown to affect spatial ability scores. For instance, Cherney et al. reported improvement of spatial abilities in undergraduate students exposed to a 1-h video game training session using Nintendo Wii or GameCube consoles (10). In contrast, by Timm et al. found that a 10-h training session with the popular video game, Tetris, had no effect on spatial ability measured by mental rotation test scores (MRT) (11). In other studies, chess playing was found to be a factor related to spatial abilities and general reasoning. A combination of above-average general intelligence and strong spatial ability was found to be present in gifted young Belgian chess players (12). Cognitive benefits of chess training lessons were also established in children. Primary school students provided with chess lessons improved their school performance in math and Romanian language (13).

To detect possible effects of highly-visual short-term anatomy canine osteology or dissection labs exposure in spatial and general non-verbal reasoning abilities and, using this short-term training concept, the present study utilized two standard tests designed to measure such abilities: GVVT and AMPT.

The hypotheses tested in this preliminary study were: (1) There is a detectable increase in spatial and general non-verbal reasoning scores in first-year female DVM students after focused exposure to short anatomy trainings in basic canine osteology or dissection; (2) There is a positive correlation between spatial ability and general non-verbal reasoning ability scores of students receiving these trainings.

## Materials and methods

Two previously validated testing tools were used to measure spatial and general non- verbal reasoning abilities in first-year veterinary medical students. The tests required 20 total minutes to complete and were delivered in an online platform provided by the University of California, Davis, school of veterinary medicine.

Participation in this experiment was voluntary. The study was considered exempt from formal review by the University of California Institutional Review Board.

### The tests taken by participants

1. Guay’s visualization of views test: adapted version (GVVT): this test measures spatial ability. The test measures the ability to correctly recognize 3-D objects viewed from different positions. It includes 24 questions, is timed at 8 min to complete, and is a modified and validated version of The Purdue-Visualization of Views Test. Questions on this test show rotated images of 3-D objects suspended in a transparent cube. Individuals being tested must identify the correct corner of the cube from which a virtual picture of the suspended object was taken. The picture of the suspended object is shown above the cube in each question. Incorrect answers incur a penalty of 1/6 of a point, making the possible range of scores −4 to 24 (9).
2. Raven’s advanced progressive matrices test, short form (APMT): this test measures general non-verbal reasoning ability. This 12-question, 12- min test is a sub-set of the original full-length Raven’s Advanced Progressive Matrices Test, which was validated by Bors & Stokes. The test requires correct identification of the missing pattern in a logical design of diagrams, from a set of 8 choices. Individuals tested are not penalized for incorrect answers, such that scores fall between 0 and 12 (5, 6).

### Osteology group (OG): 4 h

Ten first-year female veterinary medical students with no training in canine osteology, ages between 22 and 34 years old, voluntarily participated in this experiment. First-year University of California (UC) Davis veterinary medical students learn canine osteology in laboratory sessions of block VET403 Musculoskeletal of the veterinary medical curriculum. The volunteer students participated in an extracurricular short basic osteology training. This extracurricular activity took place before the musculoskeletal block (where canine osteology is covered) occurred in the DVM curriculum.

The anatomy sessions were provided over a total time of 4-h in 3 consecutive days. This duration and timing approximate time spent in osteology training by veterinary students with each new body region reached. The sessions covered anatomic features of the canine appendicular (thoracic and pelvic limb) skeleton.

#### 1st day (1 h)

After an introduction, using bone boxes that each contained a complete, disarticulated canine skeleton, each student had to identify the scapula, humerus, radius and ulna. Students were also asked to identify the location the bones within a provided articulated canine skeleton and to see if they could determine if an isolated bone was from a left or the right limb. A set of landmarks, bone processes and apophysis (growth plates) was then identified with instructor assistance in each one of the bones.

#### 2nd day (1.5 h)

After an introduction, using bone boxes, students were to identify the following bones of the skeleton manus (front foot): carpal, metacarpal, and phalanges of the digits. They were also to identify general features of the canine pelvis. Students also were to identify the location the bones within the articulated canine skeleton and were to determine if an isolated carpal bone belonged to the left or the right limb.

#### 3rd day (1.5 h)

After an introduction, using bone boxes, students were to identify the femur, tibia and fibula and features of the skeleton pedis (rear foot): tarsal, metatarsal bones and phalanges. Students were also to identify the location the bones within the articulated canine skeleton and were to determine if an isolated tarsal bone belonged to the left or the right limb.

Images of bones and structures to be found were projected in the room during the training. Three instructors assisted the students during the training. OG students had an interval of 5 days between the 1^st^ and 2^nd^ testing experience (T1 and T2).

### Dissection group (DG): 19 h

A different group of 10 first-year female veterinary medical students aged between 22 and 26 years was subjected to 19 h of musculoskeletal dissection, pro-section and palpation labs (students of the DG did not overlap with students in the OG). The 19 h were merged into the integrated block musculoskeletal (VET403) of the UC Davis DVM integrated curriculum. The students spent 10 h dissecting the muscles of the trunk, neck, thoracic and pelvic limbs. The students also studied joints, blood vessels and nerves of the thoracic and pelvic limbs, tendons, tendon sheaths, and bursae with pro-sections (5.5 h). Students also had 1.5 h of live animal palpation and 1 h of anatomy review with pro-sections before the midterm and final anatomy practical exams. Students took the spatial ability and non-verbal reasoning tests before and after they completed the total 19 h of exposure to anatomy labs. It is important to note that students in the DG were exposed canine osteology within the DVM curriculum. However, they were tested immediately before starting the dissection labs (T1). They were tested once more after finalizing the 19 h of lab exposure (T2). The DG students had an interval of about 6-weeks course-block, extending time between the T1 and T2 testing experiences.

### Statistical analysis

Descriptive statistics and a Shapiro–Wilk test were performed to evaluate the absence of normal distribution. A Wilcoxon signed rank test for non-parametric data was used to compare test scores before and after trainings. A Wilcoxon rank sum test for non-parametric data was used to compare test scores between the 2 experimental groups. A Spearman’s correlation for non-parametric data was performed to determine if spatial ability test scores (GVVT) were correlated to non-verbal reasoning test scores (APMT). The Spearman correlation ranges from −1 to 1 with 0 indicating there is no tendency for the first variable to increase or decrease as the second variable increases. Statistical software XLSTAT was used to perform the data analysis. A power analysis was performed with G*Power software version 3.1 (free download). For all analyses a *p* < 0.05 was considered significant.

## Results

Results from the Wilcoxon signed rank test for the OG showed a numeric but non-significant increase in GVVT post-training scores (*p* = 0.092), with scores of 8.01 and 11.34 pre-training and post-training, respectively. Similar results were found for the APMT, with scores of 7.44 and 8.44, pre-training and post-training, respectively, (*p* = 0.16; Table 1). These trends toward significance with a small group size of 10 may suggest likelihood of significant increases occurring in larger groups of veterinary students, e.g., the full class of 150 students. Results from the Wilcoxon signed rank test for the DG showed a numeric but non-significant increase in GVVT post-labs scores (*p* = 0.67), with scores of 11.77 and 13.28 pre-labs and post-labs, respectively. For the APMT, the increase in score was significant (*p* = 0.028), with scores of 6.8 and 10.2, pre-labs and post-labs, respectively, (Table 1). When performing the Wilcoxon rank sum test to cross compare scores between the OG and DG groups, a significant difference was found for the APMT between T1/OG (pre-testing scores for OG) and the T2/DG (post-testing scores for DG, *p* = 0.011). For the other cross comparisons between groups there was no significant difference.

**Table 1:** Mean ± SEM of first-year DVM students’ scores for the 2 tests: GVVT and APMT.

Test	OG T1	OG T2	DG T1	DG T2
GVVT	8.01 ± 2.81	11.34 ± 2.77	11.77 ± 2.58	13.28 ± 2.69
APMT	7.44 ± 0.73	8.44 ± 0.77	6.8 ± 1.05	10.2 ± 0.44 *

*p < 0.05. T1, first testing, T2, second testing (N = 10 in each group).

When performing a Spearman’s correlation within the OG and DG between GVVT and APMT scores, a positive correlation was found in all the cases, with the strongest correlation found within the DG, post lab exposure scores (+0.59).

## Discussion

Gross or macroscopic anatomy has long been considered a keystone discipline in the medical professions. This early portion of the curriculum provides the foundation for later touch-based physical examinations (palpation), diagnostic imaging (radiography, computed tomography, magnetic resonance imaging, and ultrasound), surgery (applied anatomy), and pathology (2). Studies by Provo et al., established a significant correlation between spatial ability scores and performance on anatomy examinations. These authors therefore suggested that students with low spatial ability are at increased risk of poorer academic outcome in anatomy (14). Studies by Keehner et al. found that university students with high performance in spatial and visual reasoning tests were more rapid learners of surgical laparoscopic techniques by virtual reality than those with lower performance. These authors suggested that the integration of interactive 3D image post-processing software into radiology education effectively improves radiological reasoning, diagnostic skills, and confidence as well as visual–spatial ability. They further indicated that such integration has led medical students to feel better prepared for every day clinical practice (15). Regarding the veterinary medical curriculum, Gutierrez et al. found that first-year female veterinary medical students (*N* = 43) who have progressed through 64 weeks of an integrated curriculum, which contained substantial anatomy dissection and learning experience, showed improvement of their spatial visualization ability as measured by GVVT and MRT. These same students also demonstrated improvement at week 64 in non-verbal general reasoning ability as measured by APMT, and had spatial ability scores that showed a positive correlation with non-verbal general reasoning ability scores (9). A similar finding was described for both males and females after exposure to 32 weeks of the veterinary medical integrated curriculum. Such improvements were measured by GVVT and MRT (2). Trends toward significance in each of the same endpoints in the present focused anatomic studies suggest the highly-visual and tactile learning of bone and soft tissue anatomy and features may be an important contributor to the enhanced spatial visualization ability we previously observed.

There has been a great deal of speculation concerning the possible beneficial effects of short and long-term videogame training on spatial ability. It has been widely proposed that videogames can teach a range of cognitive skills, increase attention spans and concentration, and improve spatial visualization and eye-hand coordination (16). It has been established that frequent video gamers have significantly higher abilities in adapting to new spatial challenges and remaining aware of in-game elements in dynamic environments as compared to infrequent gamers. This supports the hypothesis that video game exposure positively influences spatial cognition, likely due to the again highly-visual nature of many games that require players to navigate complex environments and process spatial information rapidly (17). Regarding short videogame trainings and improvement of spatial abilities, a training as short as one-hour on either a Nintendo Wii™ or GameCube consoles has shown improvements in mental object rotation skills in both males and females. The one-hour of video game training not only increased women’s mental MRT scores to a level similar to male scores, but also produced greater average improvement for women (10). In another study, a short videogame training of 4 h showed beneficial effects on spatial abilities as measured by MRT. The findings showed that women had overall larger gains than men and that men, as well as women, still have substantial room for improvement with continued training. It was seen as particularly noteworthy that only 4 h of computer game practice produced an improvement in MRT performance, and that longer times of practice produced higher gains (18).

Using this short-term training concept as described above, the present study was performed to determine if 4 h and/or 19 h anatomy exposure experience would positively affect spatial and non-verbal reasoning abilities in first-year female veterinary medical students. A limitation of the present preliminary study was the small number of participants from the present class who volunteered (*N* = 10 in each group), with a calculated power analysis of 0.48. Also, for this same reason, the present testing lacks a control group to determine if repeat-taking of the tests may result in a higher score on the second experience. However, this possibility may be minimized by OG and DG students having had different intervals of 5 days and 6 weeks between the T1 and T2, respectively. The aspect of small number of students in each group is a difficult one to control as participation is voluntary and students can drop the study at any time. Also, the veterinary medical curriculum is very demanding and extensive which makes it difficult to find mutually agreeable times for such trainings outside of the curricular frame for the osteology group study. Nonetheless, results suggest that a higher number of participants may lead to significant increases in scores after short anatomical trainings.

The GVVT is a test of spatial abilities, designed to determine ability to mentally recognize and manipulate 3-D objects. Raven’s APMT test is related in quantifying general non-verbal reasoning ability, through ability to identify missing patterns in complex logical designs. Scores of these two tests were positively correlated pre- and post-exposure to anatomy in both groups, providing evidence that these abilities are linked in the short term. Studies by Gutierrez et al. have previously shown long term positive correlation between spatial ability and non-verbal reasoning scores in veterinary medical students and in veterinarians working in the specialty area of diagnostic imaging (3, 9). Interestingly, scores of APMT showed a significant increase in the DG after 19-h of exposure to anatomy labs. Similar results were previously reported in first-year female students exposed to 32 and 64 weeks of the veterinary medical curriculum with 57-h and 95-h of exposure to veterinary anatomy labs, respectively (2, 9). This similar and more-focused finding at a shorter duration again suggests reasoning/logical skills increase during the exposure to the veterinary anatomy curriculum. This idea might be expanded in future experiments to determine if students with lower reasoning/logical entry-level skills may be at a disadvantage for advancing through the anatomy curriculum.

## Conclusion

This preliminary study found: (1) there was a numeric but non-significant difference in GVVT scores before and after 4 h of extra-curricular canine osteology exposure in first-year DVM students; (2) there was a numeric but non-significant difference in APMT scores before and after a 4 h extra-curricular canine osteology exposure in first-year DVM students; (3) there was a numeric but non-significant difference in GVVT scores after 19 h of curricular exposure to anatomy labs in first-year DVM students; (4) there was a significant increase in APMT (reasoning/logical test) scores after 19 h of curricular exposure to anatomy labs in first-year DVM students; (5) there was a positive correlation between spatial-ability tests scores and general-non-verbal reasoning test scores. Future studies are needed with higher numbers of students and, preferably, more groups with different hours of exposure to veterinary anatomy. It will be also interesting to expand the research to the concept of the importance of pure reasoning/logical skills as an advantage for success in the veterinary anatomy curriculum. For this purpose, a set of non-verbal reasoning (logical) tests would be a major focus. Future studies might also evaluate subgroups such as pre-veterinary students, who may be more likely to volunteer for training and testing.

## Data Availability

The raw data supporting the conclusions of this article will be made available by the authors, without undue reservation.
